# Cardamonin ameliorates neuroinflammation in Parkinson’s disease by regulating NF-κB signaling

**DOI:** 10.1186/s41065-026-00660-3

**Published:** 2026-03-09

**Authors:** Yezi Xia, Yinwei Zhang, Ying Li, Xiaojing Li, Yaling Wu, Qi Yao

**Affiliations:** Department of Geriatrics, The First Affiliated Hospital of Ningbo University, Zhejiang Province, Ningbo, 315000 P.R. China

**Keywords:** Parkinson's disease, machine learning, NF-κB, cardamonin

## Abstract

**Background:**

Neuroinflammation can accelerate neurodegeneration in Parkinson’s disease (PD). This study aims to identify biomarkers related to PD inflammation and investigate the function of cardamonin (CD) on microglia and neurons.

**Methods:**

The differentially expressed genes (DEGs) in PD were screened out. Inflammation-related genes (IRGs) were obtained from the GSEA and GeneCards databases. The protein-protein interaction network was constructed, and three machine learning algorithms, least absolute shrinkage and selection operator (LASSO) regression analysis, random forest (RF) and support vector machine (SVM) were used to screen the core IRGs in PD pathogenesis. Potential drugs for core genes were screened through the ITCM and HERB databases, and the binding activity between the core targets and the drugs was verified by molecular docking. BV2 cells were induced with MPP + to construct a PD model. CCK-8 assay, flow cytometry, enzyme-linked immunosorbent assays, qPCR and western blotting were conducted to explore the function of CD on the activation of microglia and injury of neurons..

**Results:**

One thousand one hundred eighty-three DEGs and 518 IRGs were obtained, and 31 genes were in the intersection, among which v-rel reticuloendotheliosis viral oncogene homolog A (RELA, p65), a crucial component in NF-κB signaling, was upregulated in the PD samples and demonstrated good diagnostic efficacy. CD had good binding ability with RELA. CD significantly improved the viability, repressed apoptosis, reduced the production of pro-inflammatory factors (TNF-α, IL-6 and IL-1β) of BV2 cells, which were induced by MPP+. In addition, CD significantly inhibited the activation of the NF-κB signaling pathway and the nuclear translocation of RELA.

**Conclusion:**

CD inhibits the inflammatory response of PD induced by microglia by targeting RELA and regulating the activation of NF-κB signaling.

**Graphical abstract:**

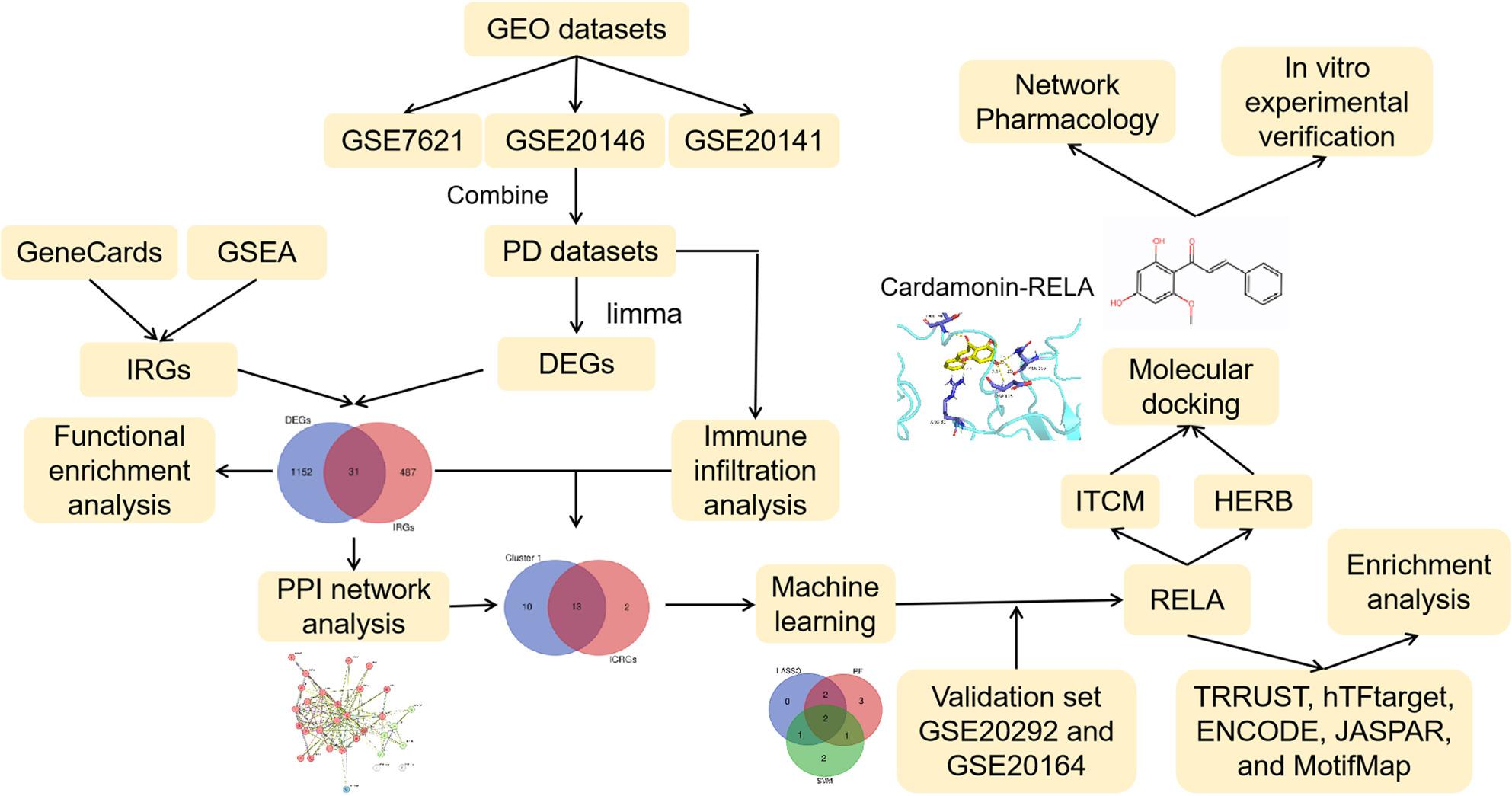

**Supplementary Information:**

The online version contains supplementary material available at 10.1186/s41065-026-00660-3.

## Introduction

Parkinson’s disease (PD) is a progressive disease caused by degeneration and death of dopaminergic neurons in the substantia nigra [1]. The clinical features of PD include bradykinesia, resting tremor, body stiffness, and changes in posture and gait; and non-motor symptoms, such as anxiety, sleep disorders and dementia, seriously affecting the quality of life of patients [2]. PD affects over 1% of people aged 65 and above, and it is predicted that the prevalence of PD will increase significantly in the following decades [3, 4]. At present, the clinical therapeutic drugs mainly target the control of motor function and the maintenance of endogenous dopamine levels, and the application of these drugs is often accompanied by side effects such as movement disorders [5–8]. Moreover, these drugs can only alleviate clinical symptoms but cannot prevent the progression of PD. Therefore, there is an urgent need to develop novel drugs.

Neuroinflammation is an immune response to pathogenic stimuli or tissue damage, mainly protecting the central nervous system (CNS) from harm and promoting tissue repair [9]. However, the severity of the injury and the persistence of the immune response may cause an imbalance in the neurochemical processes, exacerbate neuronal death, lead to the disruption of the blood-brain barrier (BBB), thereby resulting in neurological injury [10]. Microglia are the main cell type in neuroinflammatory responses [11, 12]. Although chronic neuroinflammation may not be the cause of PD, it is an important factor in the development of PD.

Cardamonin (CD, Alias: Cardamomin) is a representative member of the chalcone family, mainly extracted from *Alpinia katsumadai*. CD exerts anti-oxidative stress, anti-tumor, anti-inflammation effects [13–15]. For instance, CD can alleviate experimental colitis and related colorectal cancer [15]. CD has neuroprotective effects and alleviates cerebral ischemia/reperfusion injury by activating the HIF-1α/VEGFA pathway [16]. Pharmacological analysis has suggested that CD is a promising candidate drug for the treatment of PD [17]. However, the mechanism of CD in modulating neuroinflammation in PD has not been fully elucidated.

Based on PD-related Gene Expression Omnibus (GEO) dataset, in the present work, crucial inflammation-related genes (IRGs) in PD were screened through bioinformatics and machine learning. Natural active ingredients targeting core targets were further identified. Finally, the pharmacological effects of CD on microglia amd neurons were investigated.

## Materials and methods

### Screening of Differentially Expressed genes (DEGs) and Inflammation-Related Genes (IRGs)

GSE7621, GSE20146, GSE20141, GSE20292 and GSE20164 from the Gene Expression Omnibus (GEO) database were downloaded (Table 1). R package inSilicoMerging was applied to merge GSE7621, GSE20146 and GSE20141 datasets [18, 19]. The combined dataset was used as the exploration datasets, to screen the DEGs between PD and control samples, and the GSE20292 and GSE20164 datasets were used as the validation datasets [*P* < 0.05 and log_2_fold change (FC) > 0.585]. The gene sets HALLMARK_INFLAMMATORY_RESPONSE.v2025.1.Hs, from Gene Set Enrichment Analysis (GSEA) database, and the genes associated with inflammation from GeneCards database (accessed on 18 Aug 2025) were merged, which were regarded as the IRGs. DAVID database (https://davidbioinformatics.nih.gov/summary.jsp) was used for functional enrichment analysis (*P* < 0.05 and the *FDR* < 0.05 or 0.1) [20]. Immune cell scoring was performed on the samples in the exploration databset using the ssGSEA algorithm, provided by BioBean (Sheng-Xin-Dou-Ya-Cai, http://www.sxdyc.com/). Subsequently, the Spearman correlations between the genes in the intersection of DEGs and IRGs, and immune cells were analyzed.

**Table 1:** The datasets used in the present work

Data set	Platforms	Control	PD
GSE7621	GPL570	9	16
GSE20146	GPL570	10	10
GSE20141	GPL570	8	10
GSE20292	GPL96	29	22
GSE20164	GPL96	10	12

### Screening of the core targets in the inflammation of PD pathogenesis

The protein-protein interaction (PPI) network was constructed using the STRING database (https://cn.string-db.org/). The core genes in the network were further screened using three machine learning algorithms [least absolute shrinkage and selection operator (LASSO) regression, random forest (RF), support vector machine (SVM)]. LASSO regression was performed using the “glmnet” package in R software, RF was performed using the “randomForest” package, and SVM was performed using the “e1071” and “msvmRFE.R” packages.

### Pharmacological analysis of the targets of v-rel reticuloendotheliosis viral oncogene homolog A (RELA, p65)

From hTFtarget database (https://guolab.wchscu.cn/hTFtarget/#!/), ENCODE database (https://maayanlab.cloud/Harmonizome/dataset/ENCODE+Transcription+Factor+Targets), JASPAR database (https://maayanlab.cloud/Harmonizome/gene_set/RELA/JASPAR+Predicted+Transcription+Factor+Targets), MotifMap database (https://maayanlab.cloud/Harmonizome/gene_set/RELA/MotifMap+Predicted+Transcription+Factor+Targets) and TRRUST database (https://www.grnpedia.org/trrust/), the downstream targets of RELA were retrieved. Then the targets in the intersection were obtained. Subsequently, these targets were visualized using cytoscope v3.9.1 software. The active ingredients were searched in ITCM database (http://itcm.biotcm.net/) and HERB2.0 database (http://herb.ac.cn/v2), and the drugs in the intersection were collected. These drugs were further screened based on pharmacological parameters including drug-likeness, oral availability (> 30%), and BBB permeability (> 0) in the HERB2.0 database and the TCMSP database (https://tcmsp-e.com/index.php).

### Molecular docking

From the PubChem database (https://pubchem.ncbi.nlm.nih.gov/), the SDF file of the small molecule drugs were downloaded. Then the 2D structure of the compound was imported into the Chem3D software to minimize the energy (MM2 force field) and converted into 3D structure. Subsequently, in AutoDock tool 1.5.6, hydrogen was added and protonation was performed, and the torque was set in the software. Then the structure was saved as a PDBQT ligand file. The crystal structure of RELA (1NFI) was downloaded from RCSB PDB database (https://www.rcsb.org/). Water molecules, co-crystalline gametes and ions, etc. were removed using PyMol v2.4.0 software, and hydrogen and charge were added in AutoDock Tool 1.5.6 software. Finally, the structure was saved as a PDBQT protein receptor file. The PDBQT structures of the receptor and ligand were imported into the AutoDock Tool 1.5.6 software to construct the docking mating box. Molecular docking was performed using AutoDock Vina software and the docking affinity was calculated. The complexes were visualized using PyMol v2.4.0 software (3D) and LigPlus v2.3 software (2D).

### Cell culture

BV-2 and HT22 cells (Shanghai Institute of Cell Biology, Chinese Academy of Sciences, Shanghai, China) were cultured in Dulbecco’s modified Eagle’s medium (DMEM, Gibco, New York, NY, USA) containing 10% fetal bovine serum (FBS, Gibco), 100 U/mL streptomycin, 100 µg/mL penicillin (Gibco) and incubated at 37 °C with 5% CO_2_. The full length of RELA cDNA was inserted into the pcDNA3.1 vector (RiboBioCo., Guangzhou, China). Subsequently, the pcNDA3.1 empty plasmid and pcDNA3.1-RELA vector plasmid were transfected into the BV2 cell line using Lipofectamine^®^3000 (Invitrogen, Carlsbad, CA, USA), respectively. In vitro PD model was established using BV2 cells treated with 1-methyl-4-phenylpyridinium-iodide (MPP+, 500 µM, Sigma-Aldrich, St. Louis, MO, USA). CD (> 95% purity, Solarbio, Beijing, China) was dissolved in dimethyl sulfoxide (DMSO) and diluted it to the working concentration. NF-κB inhibitor BAY 11-7082 (2 µM; Solarbio, Beijing, China) was used to inhibit the NF-κB signaling. Corning Transwell polycarbonate membrane inserts (Corning Costar Corp, USA) were used to establish the co-culture system of BV-2 and HT22 cells.

### Cell viability assay

Cell viability was determined using Cell Counting Kit-8 (CCK-8, Beyotime, Shanghai, China). BV2 cells were seeded into 96-well plates at a density of 3 × 10³ cells/well and cultured at 37 °C for 24 h. Then, the cells were treated with 0, 1, 2, 5, 10, 20 µM CD and 500 µM MPP + for 24 h, add 10 µL of CCK-8 reagent was added into each well, and the cells were further cultured at 37 °C for 2 h. Then the absorbance of the sample at 450 nm was measured using a microplate spectrophotometer (Thermo Fisher Scientific, Waltham, MA, USA).

### Lactate Dehydrogenase (LDH) analysis

BV Cells were seeded into 96-well plates at a density of 3 × 10^4^ cells per well. When the cells grew to approximately 70% confluency, CD and MPP+ were added and the cells were incubated for 24 h. Then, according to the manufacturer’s protocol instructions, the LDH level released in the culture medium was determined using the LDH assay kit (Beyotime).

### Quantitative real-time PCR (qPCR)

Total RNA was extracted from BV2 cells using TRIzol reagent (Takara, Dalian, China). RNA was converted to cDNA using PrimeScript™ RT Master Mix (Perfect Real Time) (Takara). qPCR was performed using the TB Green^®^ Premix Ex Taq™ II (Takara) with the primers. The sequences of primers used were listed below: *Rela*: forward: 5′‑GAGACCTGGAGCAAGCCATT-3′ and reverse: 5′‑CTGTCACCTGGAAGCAGAGG‑3′; *inos* forward: 5′‑CTGATGTTGCCATTGTTGGTG‑3′ and reverse: 5′‑CTTTGACGCTCGGAACTGTAG‑3′; *Cox2* forward: 5′‑ CAGTTTATGTTGTCTGTCCAGAGTTTC-3′ and reverse: 5′-CCAGCACTTCACCCATCAGTT-3′; *Gapdh*: forward: 5′-GAAGGTCGGTGTGAACGGAT-3′ and reverse: 5′-CAATCTCCACTTTGCCACTGC-3′ The primers were synthesized by Sangon Biotech (Shanghai, China).

### Apoptosis assay

Cell apoptosis was detected using the Annexin V-FITC Apoptosis Detection Kit (Beyotime). BV2 cells were washed twice with pre-cooled phosphate buffer saline (PBS). Then the cells were resuspended in the binding buffer. The cell suspension was stained with annexin V-FITC and propidium iodide (PI) and placed in the dark at room temperature for 15 min. After the cells were washed with the binding buffer, the apoptosis of the cells was detected by flow cytometry within 1 h.

### Enzyme-Linked Immunosorbent Assay (ELISA)

The supernatants of BV2 cells were collected. Subsequently, according to the manufacturer’s protocol, the levels of TNF-α (PT512), IL-1β (PI301), and IL-6 (PI326) in the cell supernatant were detected using the corresponding ELISA kit (Beyotime). The absorbance of the sample was measured using microplate spectrophotometer (Thermo Fisher Scientific, Waltham, MA, USA).

### Western blot assay

The protein was added to the loading buffer and then boiled at 100 °C for 10 min for denaturation. Equal amounts of protein (30 µg per lane) were separated by sodium dodecyl sulfate polyacrylamide gel electrophoresis, and transferred onto polyvinylidene fluoride (PVDF) membranes (Millipore, Bedford, MA, USA) and blocked with 5% skim milk. Then the membrane was incubated overnight with the primary antibodies (Cell Signaling Technology, MA, USA) at 4 °C, including anti-NF-κB p65 (#4764, 1:500), anti-phospho-NF-κB p65 (Ser536) (#3033, 1:500), anti-phospho-IκBα (Ser32) (#2859, 1:500) anti-IκBα (#9242, 1:500), anti-Lamin B1 (#13435, 1:500) and anti-β-actin (#4970, 1:5000). Next, the membranes were incubated with the membrane with anti-rabbit IgG, HRP-linked antibody (#7074, 1:5000, Cell Signaling Technology, MA, USA). Finally, visualization was performed using the hypersensitive ECL chemiluminescence kit (Beyotime), and the intensity of protein bands was quantified using Image J software (NIH, Bethesda, MD, USA). β-actin and Lamin B1 were regarded as controls. To evaluate the effect of CD on the nuclear translocation of p65, proteins in the cytoplasm and nucleus were extracted respectively using NE-PER™ nuclear and cytoplasmic extraction reagents (78835, Thermo Fisher, Rockford, IL, USA). Subsequently, the western blot experiment was conducted.

### Statistical analysis

Statistical analysis was performed using GraphPad 6.0 software (GraphPad Prism, Inc., La Jolla, CA, USA). All of the experimental data were expressed as “mean ± standard deviation”. The measurement data between the two groups were compared by independent sample t-test, while those among multiple groups were compared by one-way analysis of variance (ANOVA), followed by Tukey *post-hoc* test and correction for multiple comparisons. A *P* value < 0.05 indicates statistical significance.

## Results

### Screening DEGs in PD and IRGs

**Fig. 1:**
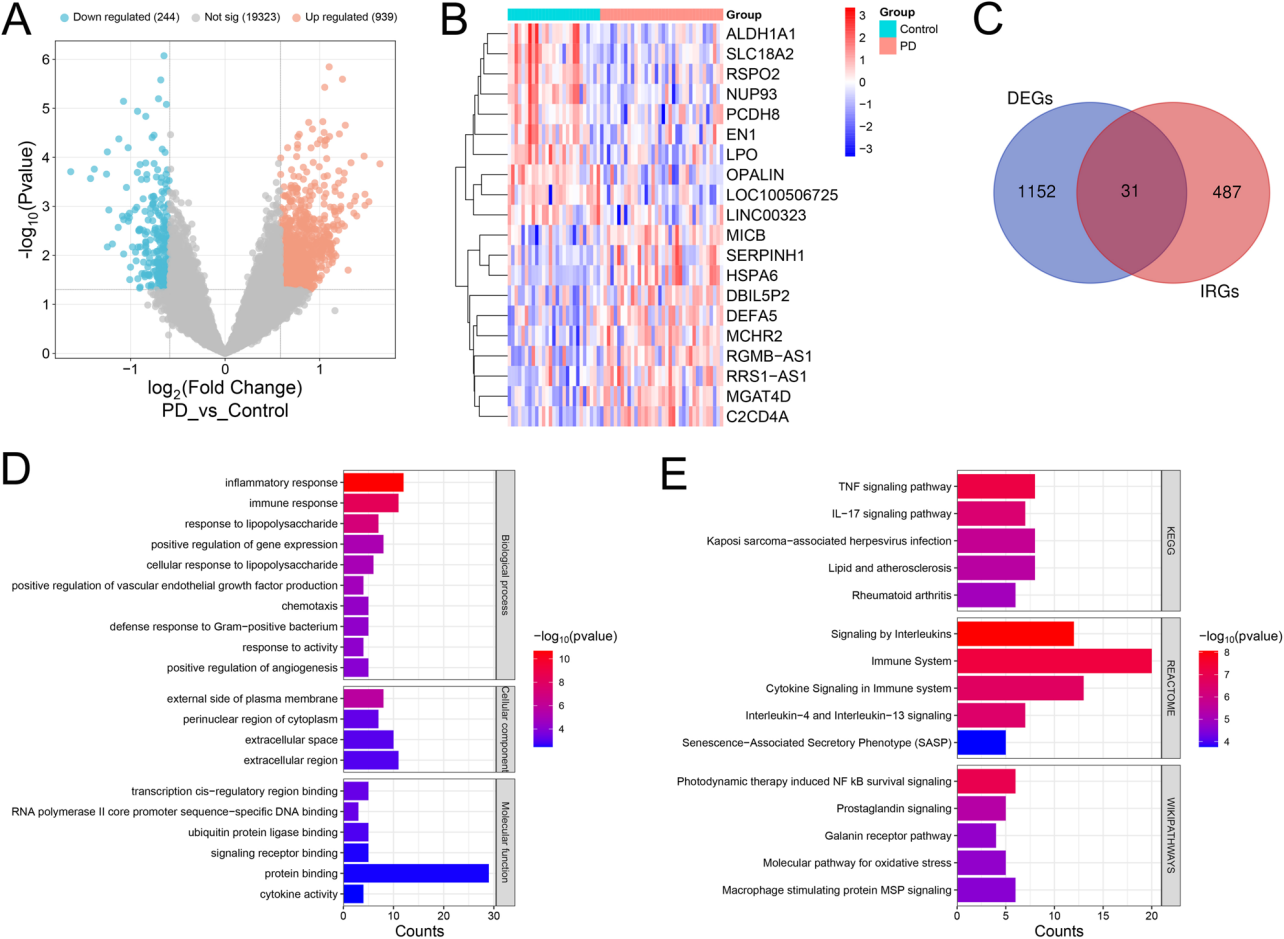
Screening DEGs in PD and IRGs. **A** The volcano plot shows results of differential expression analysis in the merged dataset. Blue dots represent down-regulated genes, orange dots represent up-regulated genes, and gray dots represent genes with insignificant differences. **B** The heat map shows the expression profiles of the top 10 genes with significantly up-regulated and down-regulated expression in the control group and the PD group. Light blue represents the control group, and red represents the PD group. **C** The Venn diagram shows the genes in the intersection of DEGs and IRGs. **D** The bar chart shows the GO enrichment analysis results of the three items of biological process, cellular component and molecular function of the genes in the intersection. **E** The bar chart shows the pathway enrichment analysis (KEGG, REACTOME and WIKIPATHWAYS) results of genes in the intersection. PD: Parkinson’s disease; DEGs: differentially expressed genes; IRGs: inflammation-related genes; GO: Gene Ontology; KEGG: Kyoto Encyclopedia of Genes and Genomes

GSE7621, GSE20146 and GSE20141, were downloaded from the GEO database, and the three datasets were merged and de-batched (Supplementary Figure 1). In the merged dataset, 1183 DEGs were obtained, including 939 up-regulated genes and 244 down-regulated genes (Figure 1A&B). Additionally, a total of 518 IRGs were obtained, and 31 genes were obtained from the intersection (Fig. 1C). GO analysis showed that these genes were mainly enriched in biological processes such as inflammatory response and immune response; cellular components such as external side of plasma membrane, perinuclear region of cytoplasm and extracellular space; molecular functions such as transcription cis-regulatory region binding, RNA polymerase II core promoter sequence-specific DNA binding (Supplementary Table 1 and Fig. 1D). The results of signal pathway enrichment analysis showed that these genes were mainly associated with TNF pathway, IL-17 pathway and NF-κB pathway (Supplementary Table 1 and Fig. 1E).

### Immune infiltration and immune-related genes in PD

**Fig. 2:**
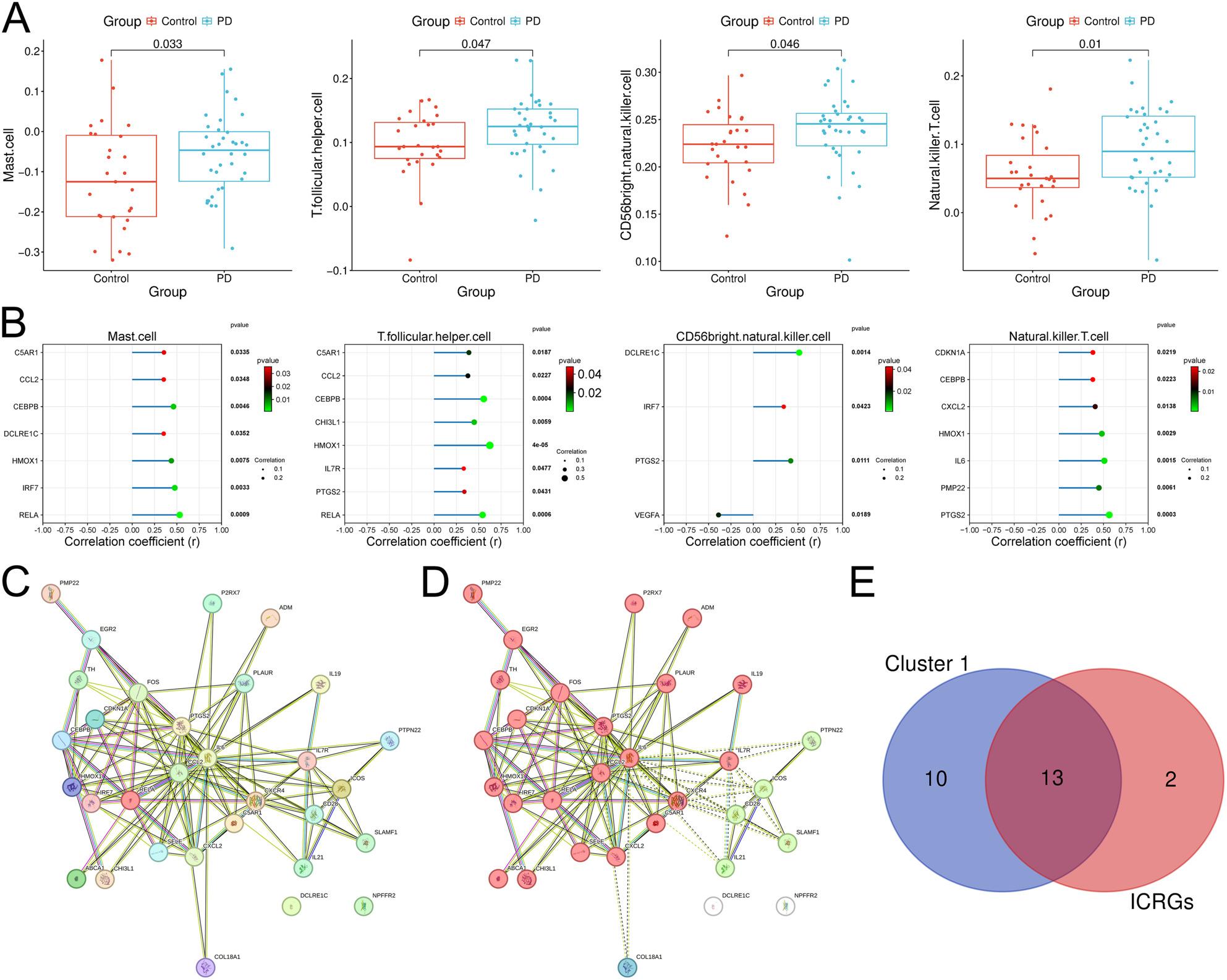
Immune infiltration analysis of the DEGs. **A** The Box plots show the differences in the infiltration levels of immune cells in the control and PD samples (Student’s t-test). **B** The lollipop chart shows the correlation coefficient between key genes and immune cell infiltration. (Spearman correlations) **C** A PPI network was constructed based on the 31 genes using the STRING database. **D** The PPI network was analyzed using the k-means clustering method. Red represents cluster 1; Green represents cluster 2; Blue represents Cluster 3; White represents unclustered proteins. **E** The Venn diagram shows the genes in the intersection of the genes in cluster 1 and ICRGs. PD: Parkinson’s disease; RGs: inflammation-related genes

The immune cell infiltration levels of all samples were evaluated by the ssGSEA method. The results showed the infiltration level of mast cell, T follicular helper cell, CD56bright natural killer cell and natural killer T cell in PD samples were significantly higher than those in the control group (Fig. 2A). Spearman correlation analysis revealed that 15 genes were significantly associated with the infiltration level of above-mentioned immune cells (Fig. 2B). The PPI network of the 31 genes was constructed using STRING database (Fig. 2C). Subsequently, three clusters were obtained through k-means clustering analysis. Cluster 1 included 23 genes and was clustered in the TNF signaling pathway. Cluster 2 included 5 genes, which were clustered in +positive regulation of alpha-beta T cell proliferation; cluster 3 included one gene, Collagen Type XVIII Alpha 1 Chain (COL18A1) (Fig. 2D). Among them, the genes in cluster 1 were regarded as key genes, and cross-crossing with immune cell-related genes (ICRGs) resulted in 13 core genes (Fig. 2E).

### Screening core genes with machine learning

**Fig. 3:**
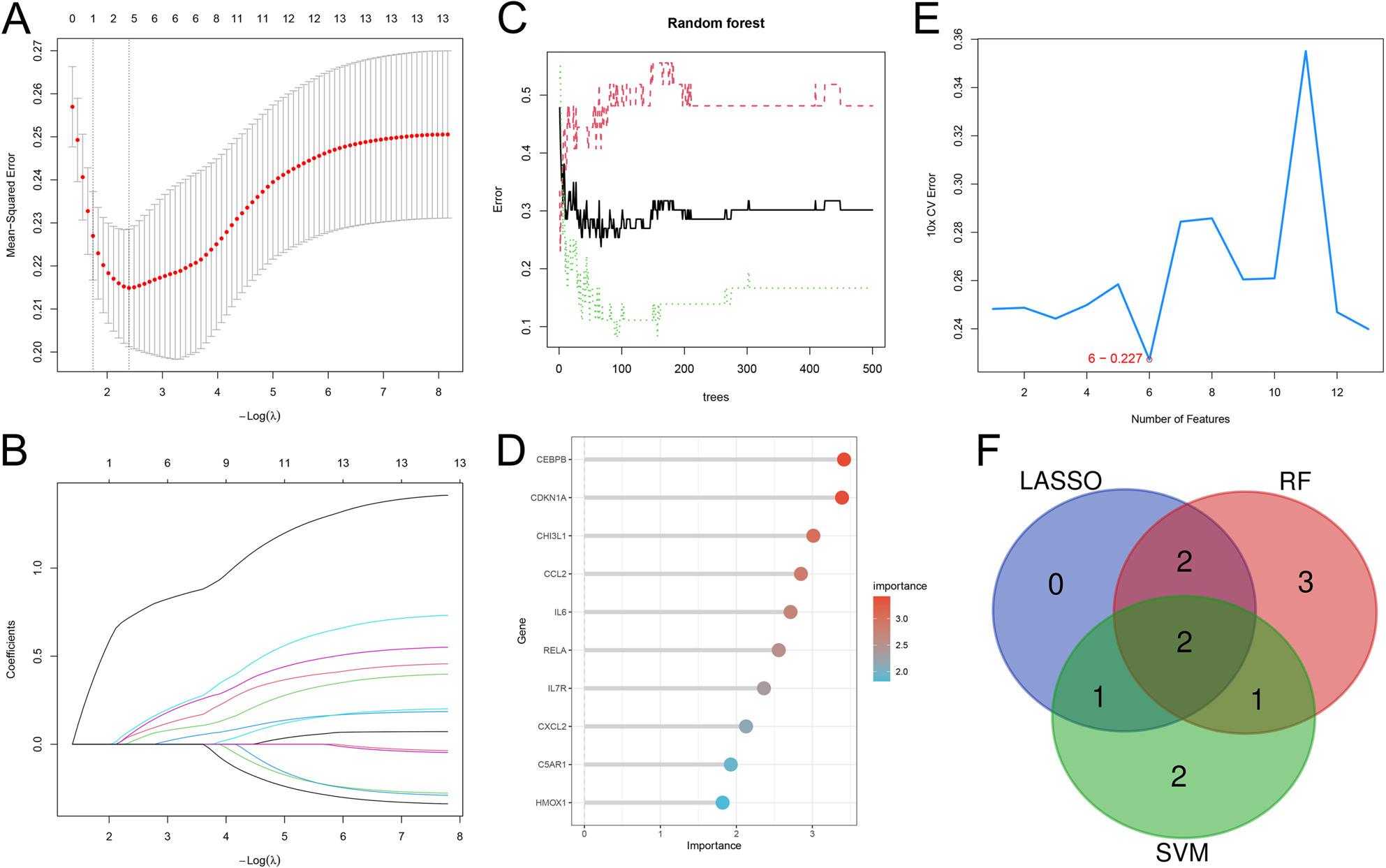
Machine learning screens core genes. **A** The coefficient distribution plots for log(lambda) sequence in LASSO. **B** The plot of gene coefficients in LASSO. **C** The relationship between the error rate of RF and the number of classification trees. **D** The top 20 relatively important genes in RF. **E** The error rate curves obtained from 10-fold cross-validation with the SVM-RFE algorithm. **F** LASSO, RF and SVM were used to obtain the Venn diagrams of the cross-genes. LASSO: least absolute shrinkage and selection operator; RF: random forest; SVM: support vector machine

**Fig. 4:**
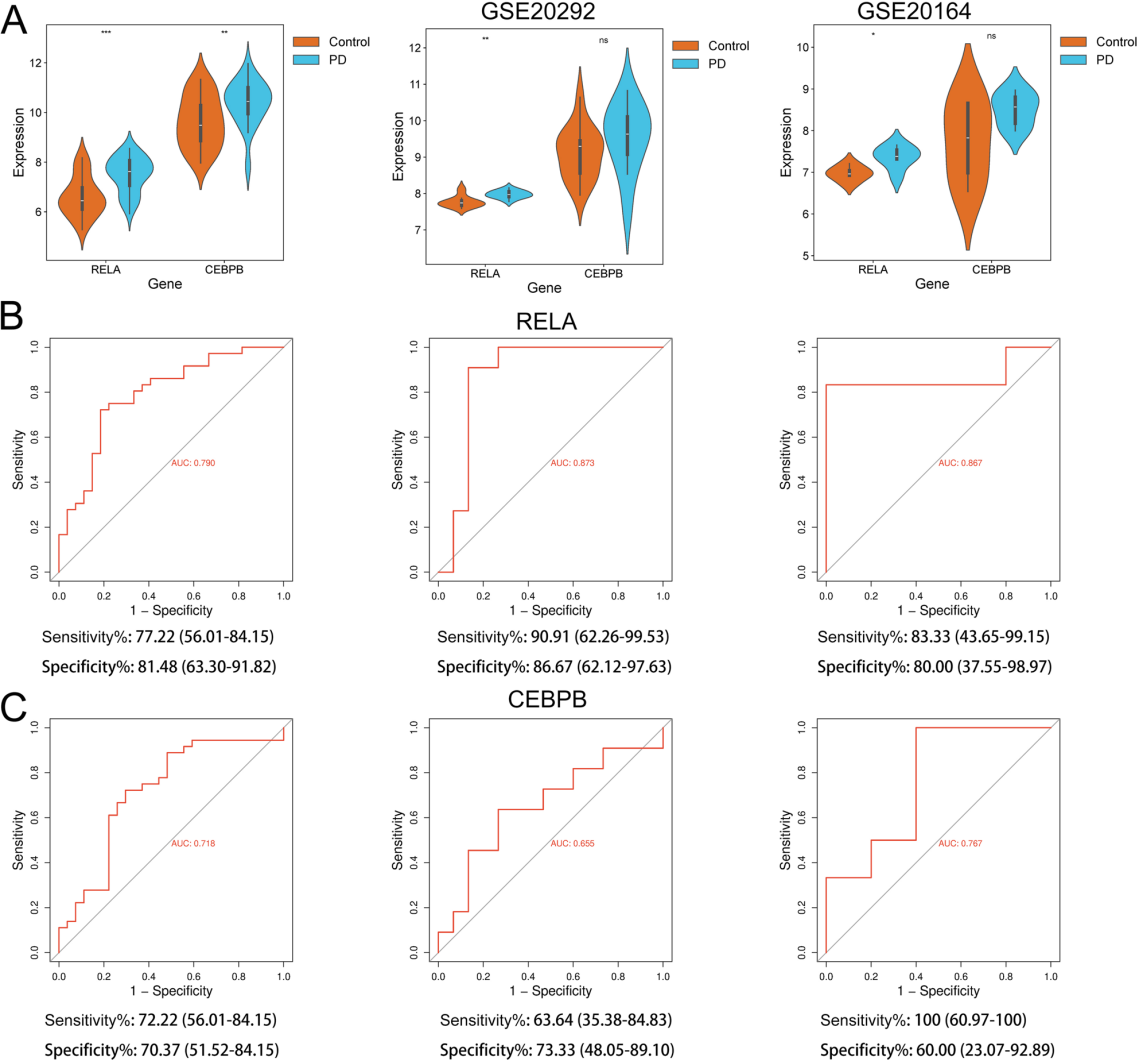
Verification of core genes. **A** The violin plot shows the expression differences of RELA and CEBPB in the control and PD samples in the merged dataset, validation datasets GSE20292 and GSE20164. **B** ROC analysis of RELA in the merged dataset, validation datasets GSE20292 and GSE20164. **C** ROC analysis of CEBPB in merged dataset, validation datasets GSE20292 and GSE20164. PD: Parkinson’s disease; ns: not significant, *P<0.05, **P<0.01 and ***P<0.001

Subsequently, the 13 core genes of PD were screened through three machine learning methods: LASSO (Fig. 3A&B), RF (Fig. 3C&D), and SVM (Fig. 3E). 5, 8, and 6 genes were obtained respectively and 2 core genes were in the intersection, RELA and CEBPB (Fig. 3F). In the merged dataset, the expression levels of RELA and CEBPB in PD samples were significantly higher than those in the control group. In the validation datasets GSE20292 and GSE20164, only the expression of RELA in the control and PD samples showed significant differences (Fig. 4A). ROC analysis showed that both RELA and CCAAT/enhancer-binding protein beta (CEBPB) had good diagnostic capabilities for PD in both the exploration dataset and the validation dataset, with area Under the curve (AUC) value greater than 0.65, and the AUC value of RELA was higher (> 0.75 in all three datasets) (Fig. 4B&C).

### Analysis of RELA’s targets and natural small molecule drugs

**Fig. 5:**
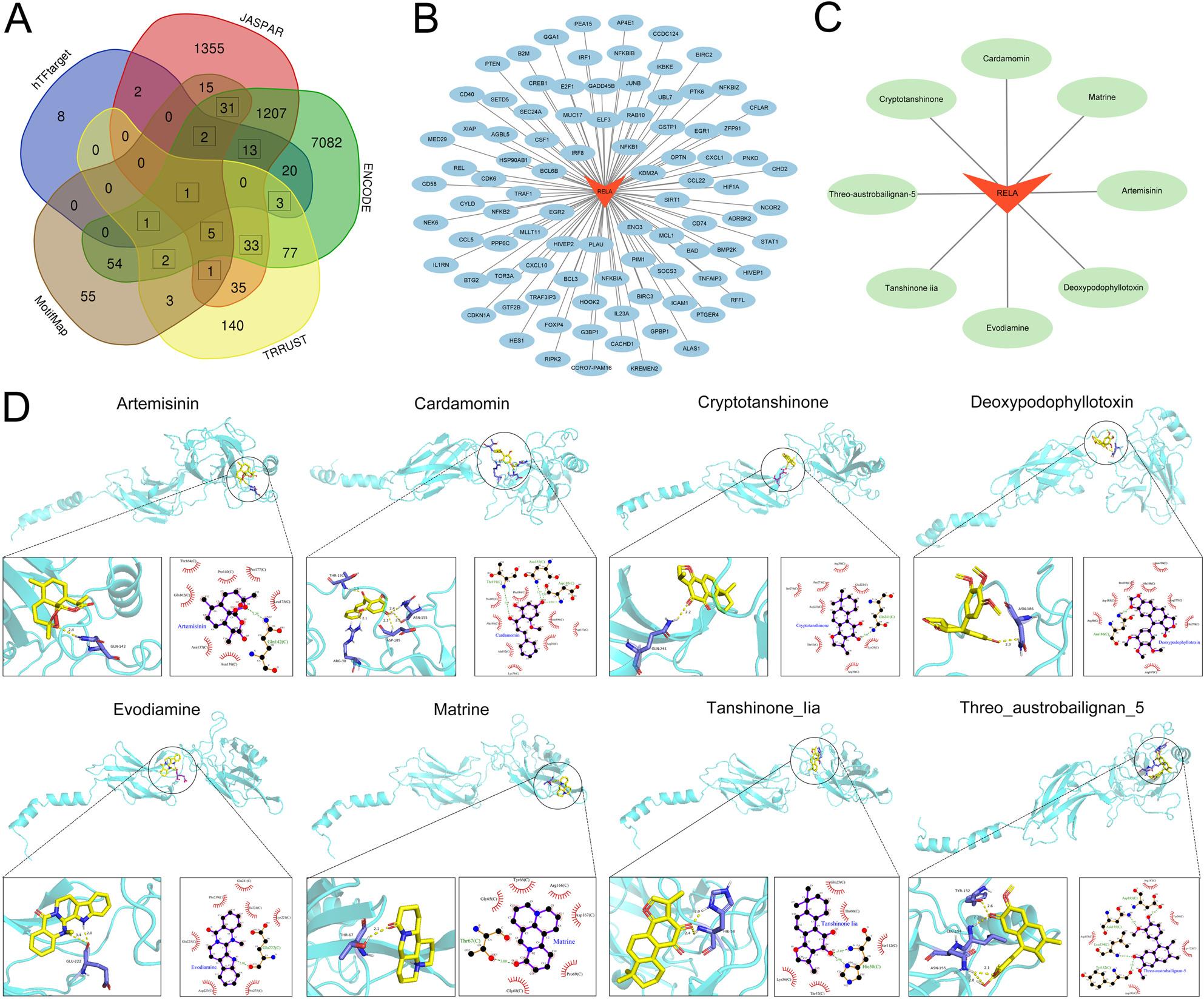
Analysis of RELA targets and natural small molecule drugs. **A** The Venn diagram of potential RELA targets in the TRRUST, hTFtarget, ENCODE, JASPAR, and MotifMap databases. **B** Cytoscope 3.9.1 software was used to construct the network diagram of RELA and targets. The red inverted triangular nodes represent the target RELA, and the blue elliptical nodes represent the target. **C** The network diagram of RELA and small molecule drugs. The red inverted triangular nodes represent the target RELA, and the green elliptical nodes represent small molecules. **D** 3D and 2D model diagrams of the binding between RELA and 8 small molecules. 3D: Blue represents RELA receptors, yellow represents gametes, purple represents bound receptor residues, and the yellow dotted line represents hydrogen bonds. 2D: Purple represents gametes, green dotted lines represent hydrogen bonds, red dandelions represent hydrophobic bonds, and yellow represents receptor residues

Potential downstream targets of RELA, as a transcription factor, were retrieved through the TRRUST, hTFtarget, ENCODE, JASPAR, and MotifMap databases, and 394, 50, 8531, 2800, and 170 targets were obtained respectively (Supplementary Table 2), and 92 targets were collected from the intersection (Fig. 5A&B). These targets were mainly enriched in inflammatory responses, apoptosis and proliferation processes, and probably participated in modualting pathways such as TNF pathway and NF-κB pathway (Supplementary Table 3). 184 and 327 active components were obtained respectively through the ITCM and HERB2.0 databases (Supplementary Table 4). Further analysis revealed eight potential PD treatment drugs: artemisinin, CD, cryptotanshinone, deoxypodophyllotoxin, evodiamine (EVO), matrine, tanshinone iia and threo-austrobailignan-5 (Fig. 5C and Supplementary Table 5). RELA had good binding activity with the 8 active molecules, and the binding energies were all less than − 6 kcal/mol. Among them, the binding ability of EVO to RELA was the strongest at -7.6 kcal/mol, followed by CD (-6.9 kcal/mol) (Fig. 5D and Supplementary Table 5).

### The dose-dependent effects of CD on the viability and apoptosis of BV2 and HT22 cells

**Fig. 6:**
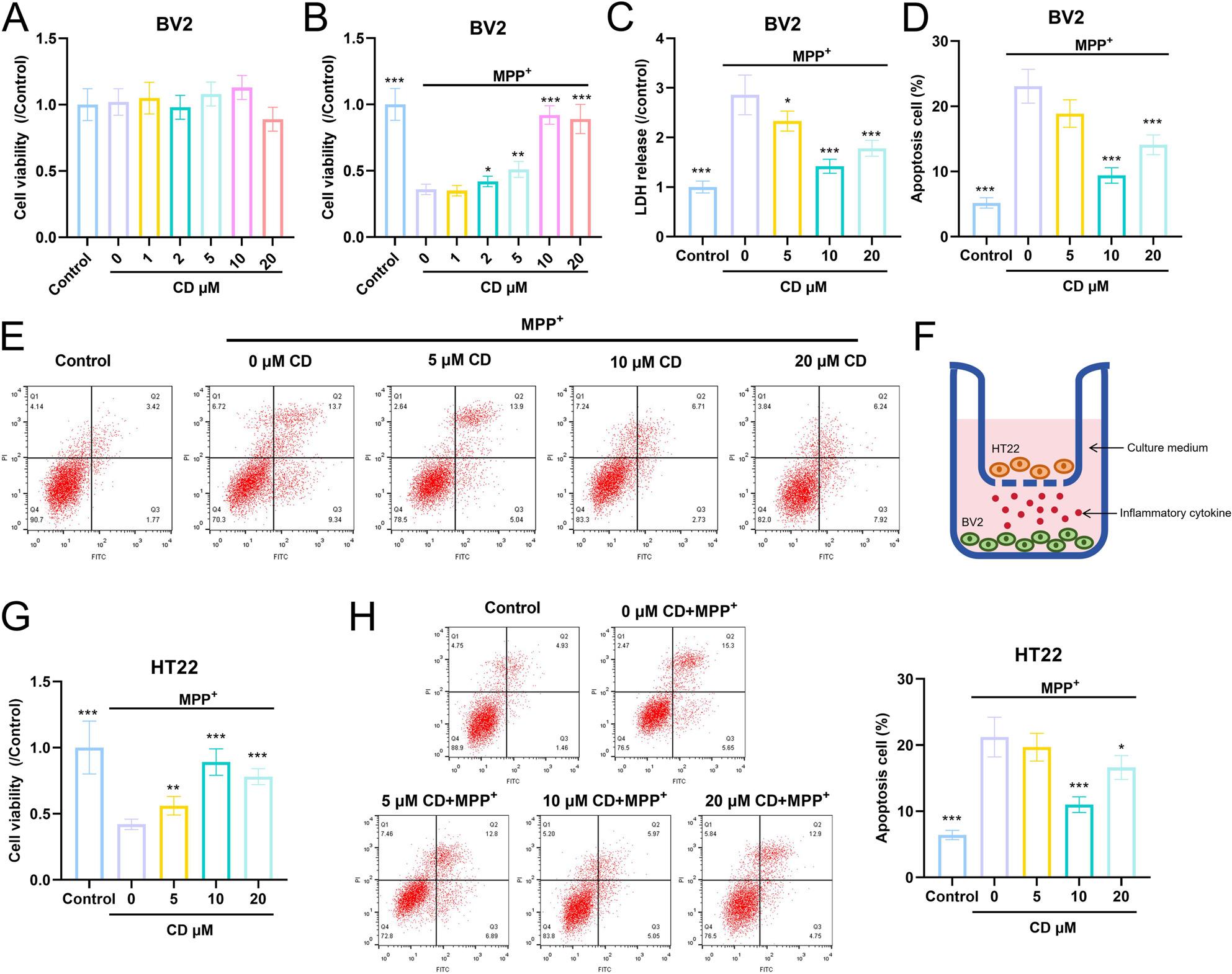
CD dose-dependently ameliorates the effects of MPP+ on the viability and apoptosis of BV2 and HT22 cells. **A**-**B** CCK-8 assay was used to analyze the effect of CD (0, 1, 2, 5, 10 and 20 μM) on the viability of BV2 cells. **C** LDH kit was used to detect the effect of CD (0, 5, 10 and 20 μM) on LDH content. **D**-**E** Flow cytometry was used to detect the effect of CD (0, 5, 10 and 20 μM) on apoptosis of BV2 cells. **F** Schematic diagram of co-culture of BV2 and HT22 cells. **G** CCK-8 assay was used to analyze the effect of CD (0, 5, 10 and 20 μM) on the viability of HT22 cells in the co-culture system. **H** Flow cytometry was used to detect the effect of CD (0, 5, 10 and 20 μM) on apoptosis of HT22 cells in the co-culture system. The measurement data among multiple groups were compared by one-way analysis of variance (ANOVA), followed by Tukey *post-hoc* test. *P<0.05, **P<0.01 and ***P<0.001 vs MPP+ group

With the increase of concentration, CD had no significant effect on the viability of BV2 cells (Fig. 6A). Compared with the control group, the viability of BV2 cells was significantly reduced under MPP+ treatment; however, as the concentration of CD gradually increased from 2 µM to 10 µM, the viability of BV2 cells gradually increased (Fig. 6B). Subsequently, this study further analyzed the effects of 5 µM, 10 µM, and 20 µM of CD on BV2 and HT22 cells. The LDH experiment results showed that the LDH content significantly decreased with the increase in CD concentration compared to the MPP+ group (Fig. 6C). The flow cytometry results indicated that the apoptosis level of BV2 cells was significantly lower than that of the MPP+ group (Fig. 6D-E). Subsequently, a co-culture experiment of BV2 and HT22 cells was conducted (Fig. 6F). The viability of HT22 cells increased with the increase in CD concentration, while the apoptosis level decreased (Fig. 6G-H).

### CD alleviates the effects of MPP + on the viability, apoptosis and inflammatory factors of BV2 cells

**Fig. 7:**
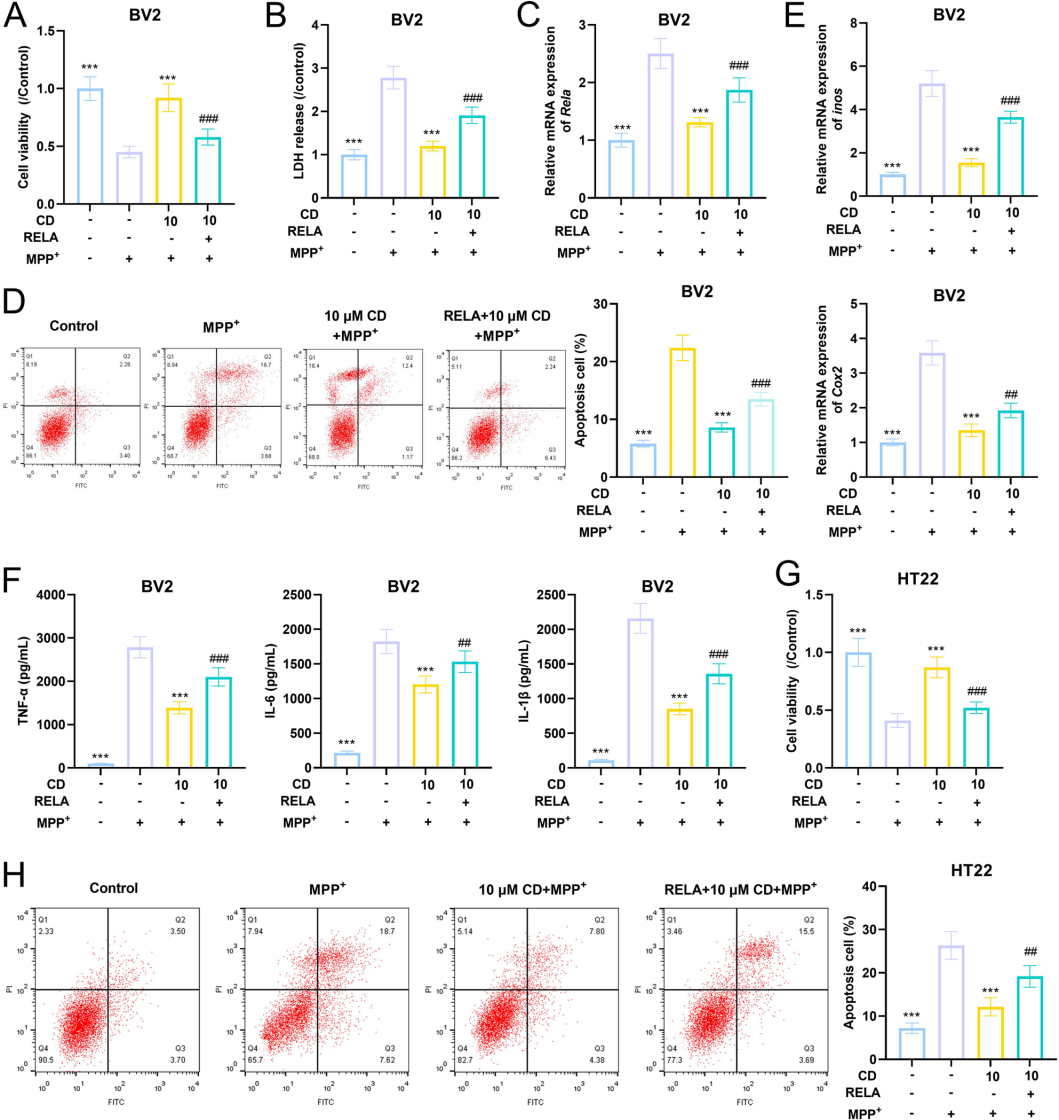
CD alleviates the effects of MPP+ on the viability, apoptosis and inflammatory factors of BV2 cells and HT22 cells through RELA. **A** CCK-8 assay was used to analyze the effect of CD on the viability of BV2 cells. **B** LDH kit was used to detect the effect of CD on LDH content. **C** qRT-PCR was used to detect the effect of CD on the transcriptional level of RELA. **D** Flow cytometry was used to detect the effect of CD on apoptosis of BV2 cells. **E** qRT-PCR was used to detect the effect of CD on the transcriptional levels of *inos* (up) and *Cox2*(down). **F** ELISA was used to detect the effect of CD on the levels of inflammatory factors TNF-α, IL-6 and IL-1β. **G** CCK-8 assay was used to analyze the effect of CD on the viability of HT22 cells. **H** Flow cytometry was used to detect the effect of CD on apoptosis of HT22 cells. The measurement data among multiple groups were compared by one-way analysis of variance (ANOVA), followed by Tukey *post-hoc* test. *P<0.05, **P<0.01 and ***P<0.001 vs MPP+ group; #*P*<0.05, ##*P*<0.01 and ###*P*<0.001 vs MPP+ + CD group; *n*=5

Compared with the control group, the viability of BV2 cells was significantly reduced under MPP+ treatment (Fig. 7A). The concentration of LDH after MPP+ treatment was significantly higher than that in the control group, and the treatment with CD significantly inhibited the increase of LDH concentration (Fig. 7B). The expression level of RELA, was significantly increased after MPP+ treatment, while CD significantly reversed this (Fig. 7C). Additionally, compared with the control group, the levels of apoptosis, pro-inflammatory enzymes (COX2 and iNOS), and pro-inflammatory factors (TNF-α, IL-6 and IL-1β) in the MPP+ treatment group were significantly increased, which were significantly inhibited in the CD treatment group (Fig. 7D-F). The results of the co-culture system experiment showed that the trends of the viability and apoptosis level of HT22 cells were consistent with those of BV2 cells (Fig. 7G-H).

### CD can inhibit the activation of NF-κB signaling

**Fig. 8:**
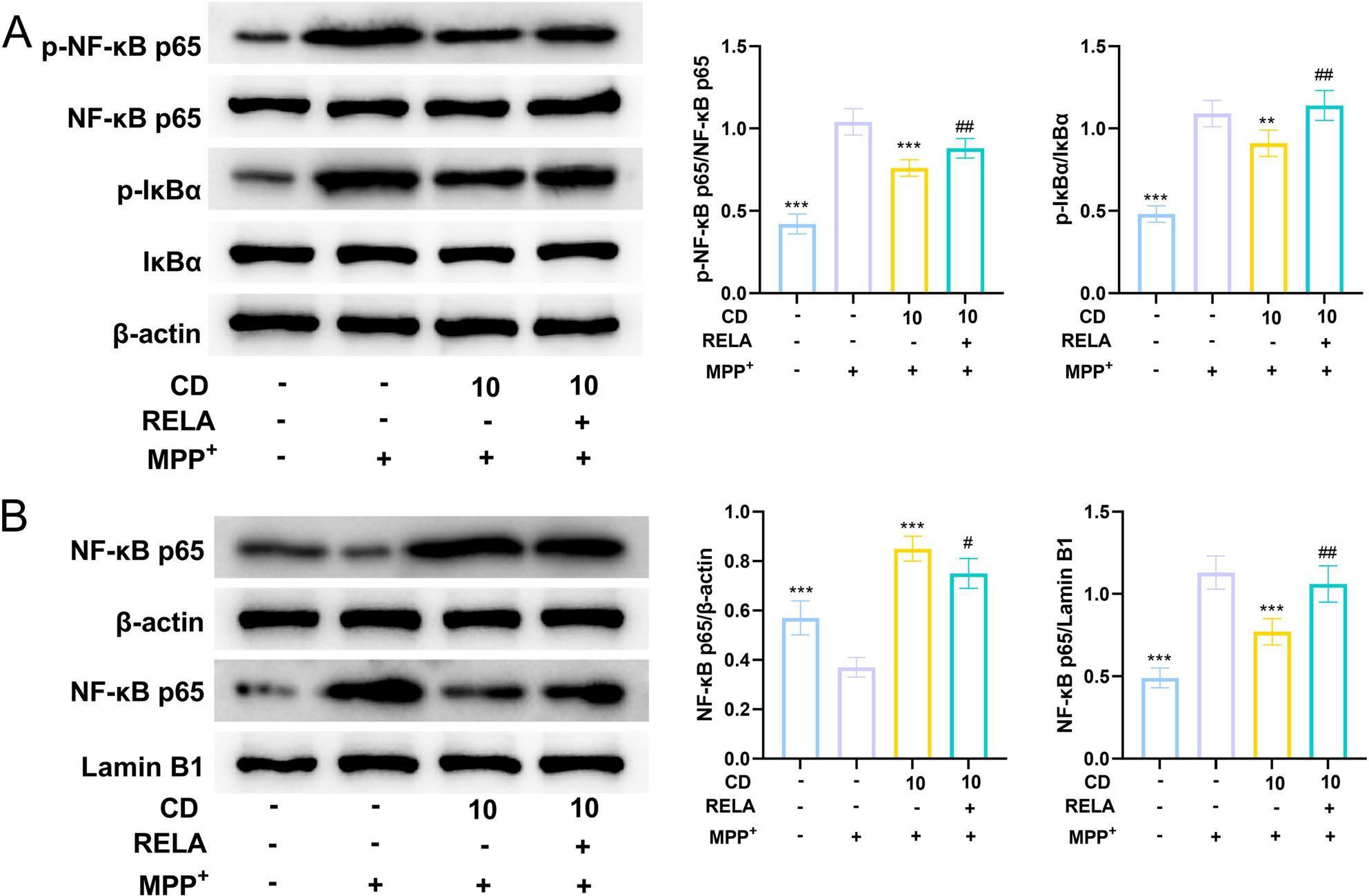
CD can inhibit the activation of the NF-κB signaling. **A** Western blot experiment was conducted to analyze the effect of CD on the phosphorylation levels of NF-κB p65 and IκB. **B** Western blot experiment was conducted to analyze the effect of CD on the levels of NF-κB p65 in the cytoplasm and nucleus. The measurement data among multiple groups were compared by one-way analysis of variance (ANOVA), followed by Tukey *post-hoc* test. *P<0.05, **P<0.01 and ***P<0.001 vs MPP+ group；##*P*<0.01 and ###*P*<0.001 vs MPP+ + CD group; *n*=5

**Fig. 9:**
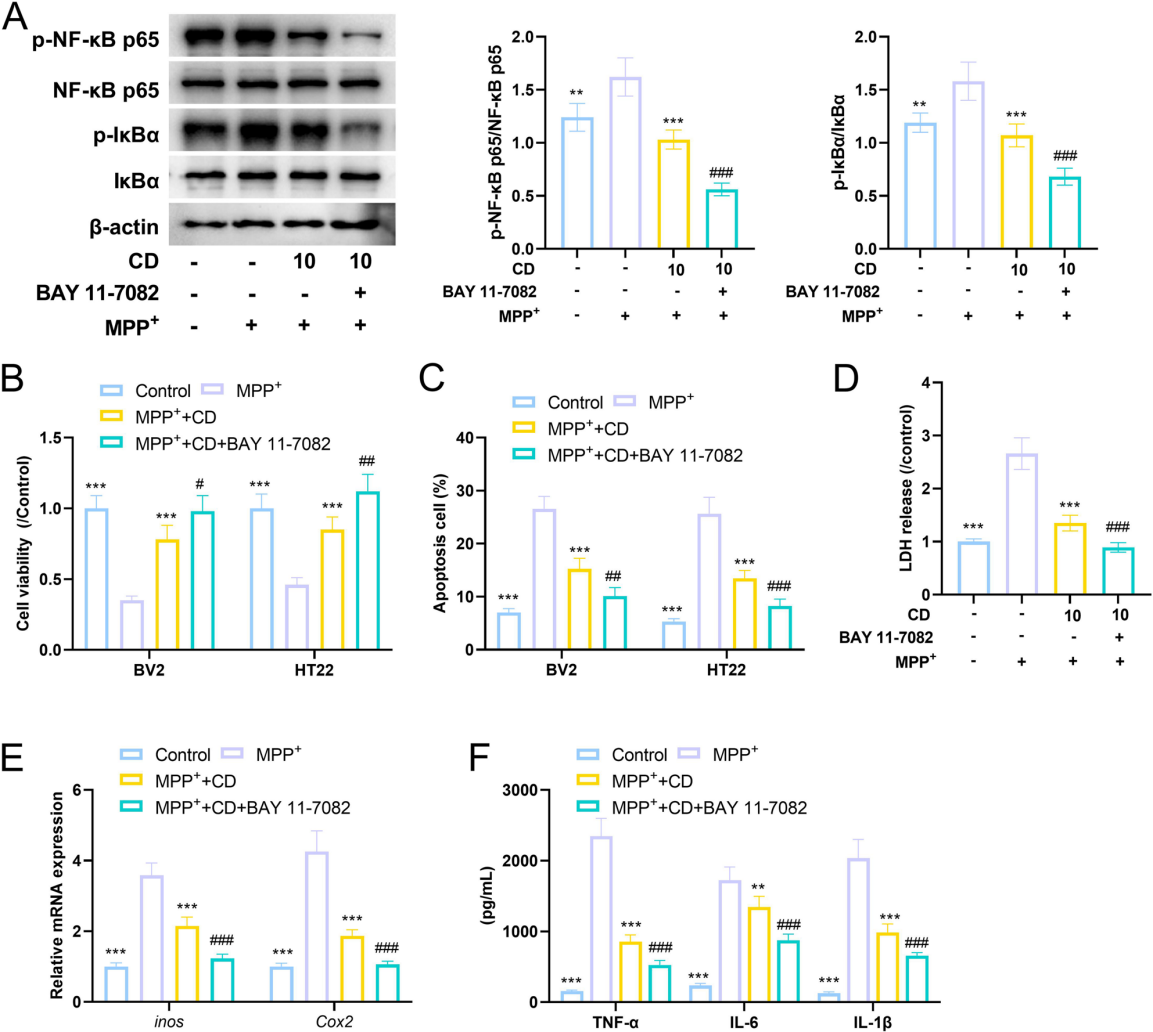
The combined treatment with CD and the NF-κB p65 signaling pathway inhibitor (BAY 11-7082) attenuates the effects of MPP+ on BV2 and HT22 cells. **A** Western blot experiment was conducted to analyze the effect of CD and BAY 11-7082 on the phosphorylation levels of NF-κB p65 and IκB. **B** CCK-8 assay was used to analyze the effect of CD and BAY 11-7082 on the viability of HT22 cells. **C** Flow cytometry was used to detect the effect of CD and BAY 11-7082 on apoptosis of BV2 cells and HT22 cells. **D** LDH kit was used to detect the effect of CD and BAY 11-7082 on LDH content. **E** qRT-PCR was used to detect the effect of CD and BAY 11-7082 on the transcriptional levels of *inos* (up) and *Cox2* (down) in BV2 cells. **F** ELISA was used to detect the effect of CD and BAY 11-7082 on the levels of inflammatory factors TNF-α, IL-6 and IL-1β. The measurement data among multiple groups were compared by one-way analysis of variance (ANOVA), followed by Tukey *post-hoc* test. **P<0.01 and ***P<0.001 vs MPP+ group；#*P*<0.05, ##*P*<0.01 and ###*P*<0.001 vs MPP+ + CD group; *n*=5

It has been reported that CD can regulate NF-κB signaling [21]. Western blot showed that the phosphorylation levels of RELA (NF-κB p65) and IκBα in the MPP+ treatment group were significantly higher than those in the control group, while the phosphorylation levels of NF-κB p65 and IκBα in the CD treatment group were significantly lower than those in the MPP+ group, and in the RELA overexpression group, their phosphorylation levels were significantly higher than those in the CD treatment group (Fig. 8A). Nuclear-cytoplasmic separation experiment showed that in the cytoplasm, the level of NF-κB p65 in the MPP+ treatment group was lower than that in the control group, while in the nucleus, the level of NF-κB p65 was higher than that in the control group; after CD treatment, the level of NF-κB p65 in the cytoplasm was higher than that in the MMP+ group, while the level of NF-κB p65 in the nucleus was lower than that in the MMP+ group; the opposite phenomena occurred after RELA overexpression (Fig. 8B). These results indicated that CD inhibited the activation of the NF-κB signaling by suppressing the phosphorylation and nuclear transfer of NF-κB p65. Notably, combination of NF-κB signaling inhibitor (BAY 11-7082) and CD showed more potent effects on the viability and apoptosis of BV2 and HT22, as well as on pro-inflammatory factors (Fig. 9A-F).

### Identification and enrichment analysis of downstream targets of CD

To further explore the functions of CD, this study utilized multiple databases to search for the other potential targets of CD. 475, 34, 105, 80 and 51 targets were obtained respectively from the TargetNet, SymMap v2, SwissTargetPrediction, HERB 2.0 and ITCM databases (Supplementary Fig. 2A and Supplementary Table 6). There were 104 common targets predicted in at least two or more databases (Supplementary Fig. 2B). Functional enrichment analysis showed that these targets were mainly enriched in G2/M transition of mitotic cell cycle, peptidyl-serine phosphorylation, insulin-like growth factor receptor signaling pathway, HIF-1 signaling pathway and IL-17 signaling pathway, etc. (Supplementary Fig. 2C&D and Supplementary Table 7).

## Discussion

The pathogenesis of PD is complex and insidious. Neuroinflammation plays an indispensable role in the development process of PD [22, 23]. In the early stage of PD, the components of immune cells undergo dynamic changes as the disease progresses, and these changes are involved in inflammatory responses, leading to neuronal degeneration and symptoms in patients [24]. In this study, two inflammatory immune-related genes were identified through differential expression analysis, ssGSEA immune infiltration analysis and machine learning, and subsequently candidate drugs for PD treatment were identified. The novelty of the present study is that it integrates bioinformatics analysis, molecular docking, and in vitro experimental validation to systematically explore the role of IRGs in PD, with a specific focus on identifying RELA as a core immune biomarker with machine learning algorithms and revealing the potential therapeutic mechanism of CD in regulating neuroinflammation through the NF-κB signaling. This study not only confirms the involvement of known inflammatory pathways in PD pathogenesis but also uncovers a novel therapeutic candidate (CD) and its specific target (RELA), providing a more precise and mechanistic basis for the development of anti-inflammatory strategies in PD treatment. Additionally, the comprehensive analysis of CD’s potential biological functions and signaling pathways through network pharmacology expands our understanding of its multi-faceted role beyond direct NF-κB inhibition, suggesting broader implications for neuroprotection in PD.

Firstly, 31 IRGs were preliminarily screened in this study. These genes are mainly involved in biological processes such as inflammatory response and immune response. Previous studies have shown that peripheral circulating CD4 + T cells in PD patients produce Th1/Th2 cytokines in response to α-synuclein [25, 26]. In the PD mouse model, CD4 + T cells contribute to the innate immune response of the CNS and the death of dopaminergic neurons [27]. The analysis of immune cells in this study found that the levels of mast cell, T follicular helper cell, CD56bright natural killer cell and natural killer T cell were significantly increased in PD. Mast cells are important contributors to the pathogenesis of the disease in the PD mouse model [28–30]. Specifically, the inflammatory mediators and chemokines [such as chemokine (C-C motif) ligand 2] in mast cells contribute to the development of PD [29, 30]. Additionally, immature NK cells (CD56) are activated in the earliest stage of PD [31, 32]. The CD56brightCD16 cells produce a large number of pro-inflammatory cytokines, such as IFN-γ, TNF-α, IL-10, IL-13 and GM-CSF [25]. NK cells can clear α-synuclein aggregates, and the systemic exhaustion of NK cells leads to neuronal degeneration in PD mouse models [33, 34]. Notably, NK cells may act as a double-edged sword in CNS diseases. On the one hand, it can induce neuron death [35], and on the other hand, it has the ability to inhibit inflammation [36]. In addition, the results of signal pathway enrichment analysis demonstrated that PD was associated with abnormalities in multiple inflammation-related signaling pathways, such as TNF signaling pathway, IL-17 signaling pathway, and NF-κB signaling pathway, etc. The previous studies have demonstrated that these signaling pathways are involved in the occurrence and development of PD [37, 38]. Collectively, our findings are consistent with these previous studies.

Subsequently, it was found that 13 IRGs were significantly associated with the aforementioned immune cells. Further, through machine learning screening, a core gene RELA was obtained. RELA encodes the NF-κB p65 protein and is a transcriptional regulatory factor involved in the transcription of various genes [39, 40]. Moreover, the significant role of the NF-κB signaling pathway in the inflammatory response of PD has also been reported by multiple studies [37, 41, 42]. To explore the drugs which potentially regulate RELA, this study retrieved natural small molecule drugs targeting RELA from multiple databases. Among them, EVO and CD were two active ingredients with relatively high binding energy for RELA. EVO is a tryptamine indole alkaloid and is the main active ingredient of *Evodia rutaecarpa*. EVO possesses multiple biological activities such as anti-tumor, cardioprotective, anti-inflammatory, antibacterial, anti-atherosclerotic and anti-Alzheimer’s disease (AD) effects [43]. In the mouse model, EVO participates in the anti-AD effect by reducing the release of inflammatory cytokines in the brain and inhibiting the activation of glial cells in the hippocampus [44, 45]. In addition, EVO has a neuroprotective effect on neuronal cells (PC12) injury induced by MPP + or H_2_O_2_ [46]. Moreover, EVO inhibits neuroinflammation caused by overactivated microglia by regulating the AKT/Nrf2/HO-1/NF-κB signaling axis [47]. However, the role of CD in PD was rarely reported. In this study, the neuroinflammatory response of PD was mimicked with in vitro model. CD treatment significantly enhanced the viability of BV2 cells, inhibited apoptosis, and inhibited the expression of pro-inflammatory mediators, reduced the injury of neurons. Additionally, CD could inhibit the expression of RELA, the phosphorylation of NF-κB p65 and IκBα, and the nuclear translocation of RELA. These results indicate that CD exerts anti-inflammatory effects by regulating the NF-κB signaling in microglia. Consistently, some previous studies have also shown that CD inhibits the activation of the NF-κB signaling in LPS-induced BV2 cells and is a potential therapeutic drug for neurodegenerative diseases [48, 49]. Furthermore, CD, in addition to having an anti-neuroinflammatory effect in neurodegenerative diseases, can also regulate nuclear factor erythroid 2-related factor 2 (Nrf2)/ Kelch-like ECH-associated protein 1 The (Keap1) signaling [48–50]. This study further analyzed the function of CD through network pharmacology and found that it could probably play an important role in modulating cell cycle, apoptosis, proliferation, epidermal growth factor receptor signaling, peptidyl serine phosphorylation and other processes, and can also regulate multiple classical pathways, such as PI3K-Akt signaling pathway, HIF-1 signaling pathway and IL-17 signaling pathway, etc. Previous studies have also reported that CD can modulate the metabolism and apoptosis of cancer cells through the mTOR/p70S6K pathway and PI3K/AKT pathway [51, 52]. From this, it can be supposed that the neuroprotective function of CD in PD may also be manifested in multiple aspects, such as neuronal survival and anti-oxidative stress, etc., which should be validated in the following studies.

It should be note that, while the current in vitro data provide strong mechanistic insights into CD’s effects on microglial NF-κB signaling, in vivo studies using PD animal models (e.g., MPTP or α-synuclein models) are necessary to confirm the therapeutic potential of CD and its ability to modulate neuroinflammation in a more physiological context. Additionally, the lack of experimental validation for CD’s BBB permeability and oral bioavailability represents a key translational gap. While we utilized established ADMET (Absorption, Distribution, Metabolism, Excretion, Toxicity) prediction databases to provide preliminary evidence supporting CD’s potential as a CNS drug candidate, these in silico results require in vivo confirmation. Future studies will prioritize addressing this by employing techniques such as in situ brain perfusion to directly assess BBB penetration, and pharmacokinetic studies in animal models to determine oral absorption efficiency, plasma half-life, and brain tissue distribution. These experiments are crucial to establish the feasibility of CD reaching its target (microglial RELA in the brain) at therapeutically relevant concentrations when administered via a clinically translatable route. Additionally, the current study focused on microglial activation and the NF-κB/RELA pathway due to the well-established role of microglia in neuroinflammation within the CNS microenvironment. Mast cells, NK cells, and T follicular helper cells are primarily peripheral immune cells, and their direct infiltration into the brain parenchyma in PD remains a topic of ongoing investigation with limited consensus. However, the inaccessibility of brain tissues of PD patients for detailed cellular infiltration analyses presents a significant practical barrier. Future studies may employ preclinical PD models to examine the crosstalk between peripheral immune cell populations and CNS microglia, and test whether CD modulate these peripheral immune cell populations as an additional mechanism of action.

## Conclusion

RELA is identified as an immune-related biomarker for PD. The relationship between CD and RELA as well as their neuroprotective functions are verified. This study imply that CD is a promising treatment approach that attenuates the neuroinflammation of PD via modulating NF-κB signaling. 

## Supplementary Information

Supplementary Material 1: Supplementary Figure 1. Merging and de-batching of three PD-related GEO datasets A. Density distribution comparison before (up) and after (down) batch removal. B. UMAP distribution comparison before (up) and after (down) batch removal. C. Comparison of data distribution before (up) and after (down) batch effect removal. Supplementary Figure 2. Identification and enrichment analysis of downstream targets of CD. A. The Venn diagram of CD targets in TargetNet, SymMap, SwissTargetPrediction, HERB 2.0 and ITCM databases. B. Cytoscope 3.9.1 software was used to construct the network diagram of CD and the targets. The green rhombus nodes represent CD, and the purple oval nodes represent the targets. C. The bar chart shows the top 10 results of the three items of biological process, cellular component and molecular function in the GO enrichment analysis. D. The bubble chart shows the top 20 items of the KEGG enrichment analysis results. Supplementary Table 1. The functional enrichment analysis results of the 31 genes in the intersection of DEGs in PD and IRGs. Supplementary Table 2. Targets of RELA predicted TRRUST, hTFtarget, ENCODE, JASPAR and MotifMap databases. Supplementary Table 3. The functional enrichment analysis results of RELA targets. Supplementary Table 4. The ingredients of ITCM and HERB 2.0 databases. Supplementary Table 5. Prediction of drug-likeness and ADMET properties of the potential drugs. Supplementary Table 6. The targets of Cardamonin in TargetNet, SymMap, SwissTargetPrediction, HERB 2.0 and ITCM databases. Supplementary Table 7. The functional enrichment analysis results of 104 targets of CD.

## Data Availability

The data used to support the findings of this study are available from the corresponding author upon request.
