# Supplementary material for: Cardamonin ameliorates neuroinflammation in Parkinson’s disease by regulating NF-κB signaling
Source: Hereditas. 2026 Mar 9;163:51. doi: 10.1186/s41065-026-00660-3 (PMC13085287; doi:10.1186/s41065-026-00660-3)

Figure 8A

p-NF-kB p65


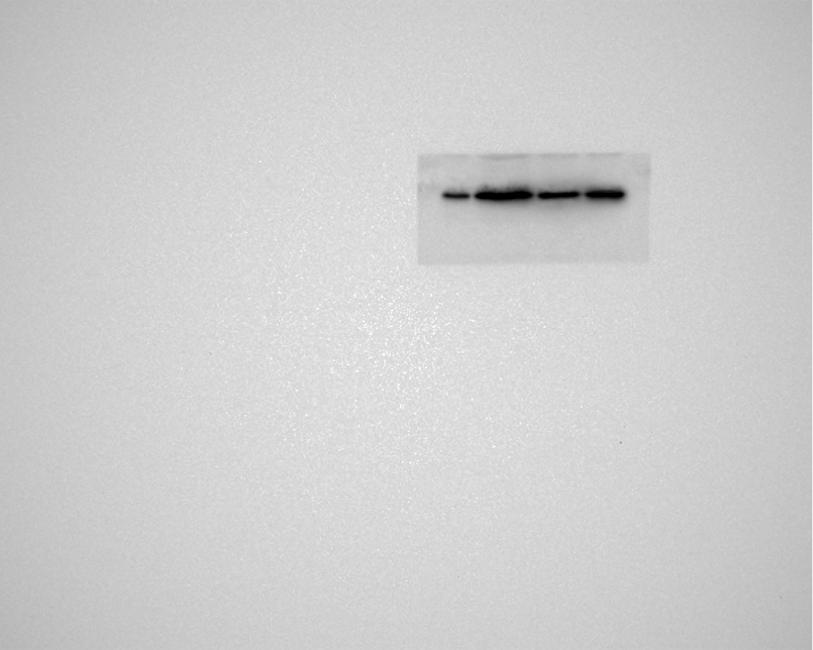


NF-kB p65


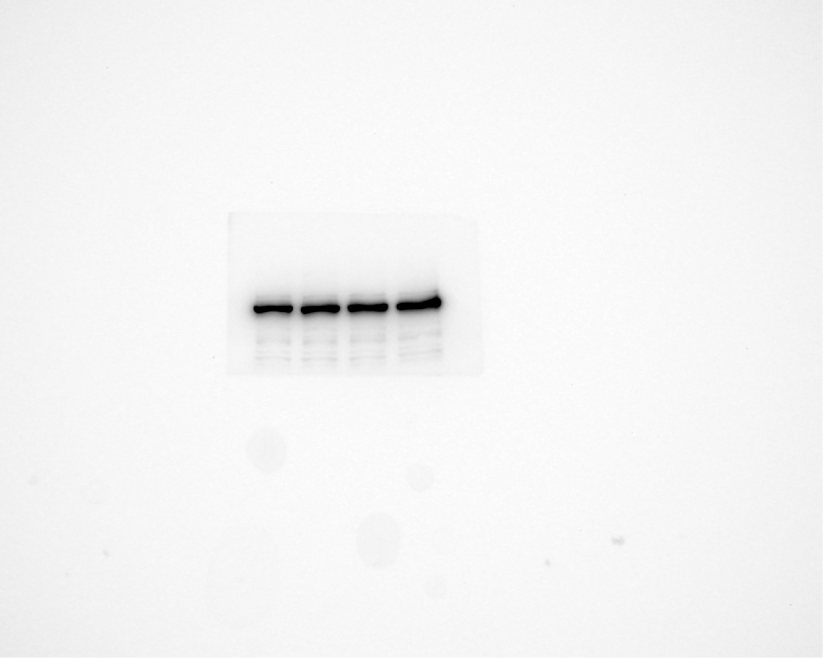


p-IκBα


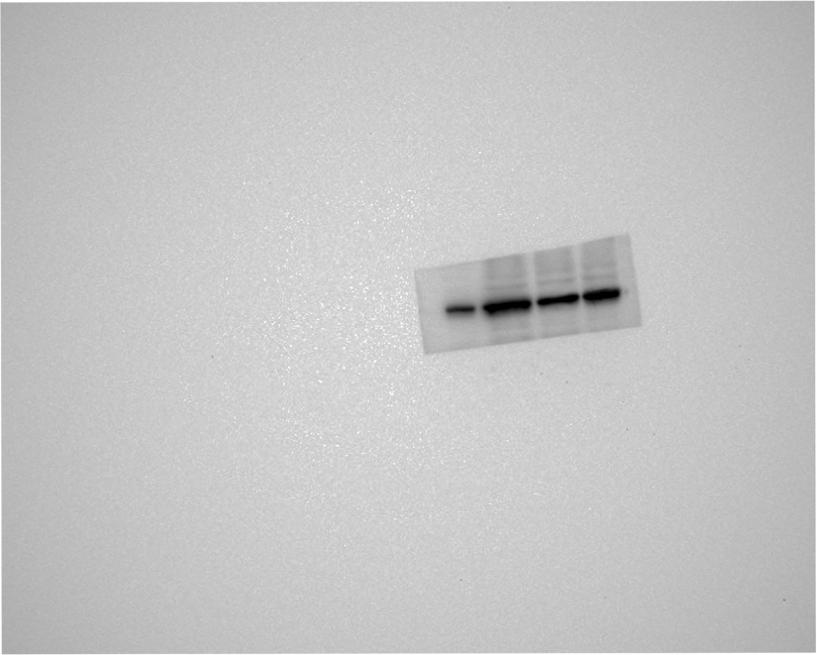


IκBα


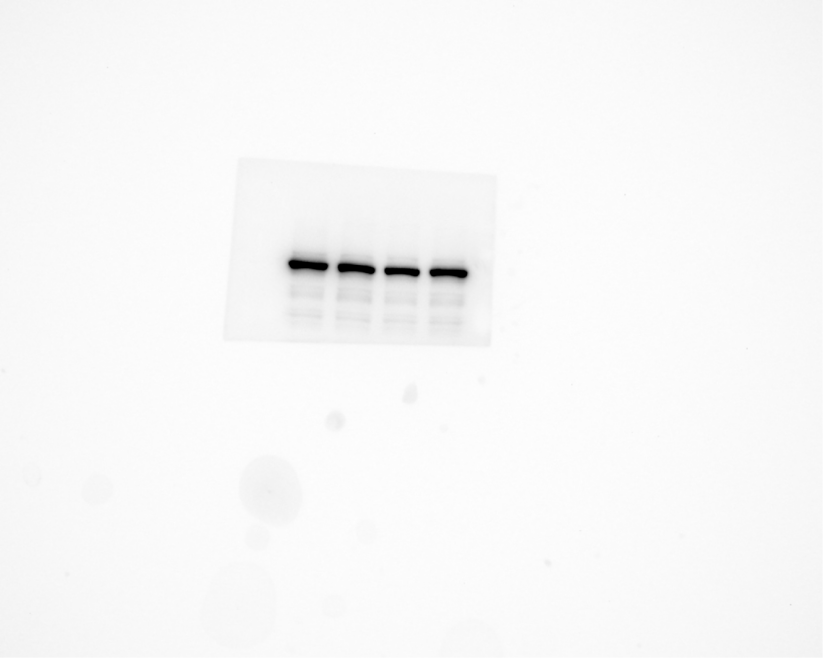


β-actin


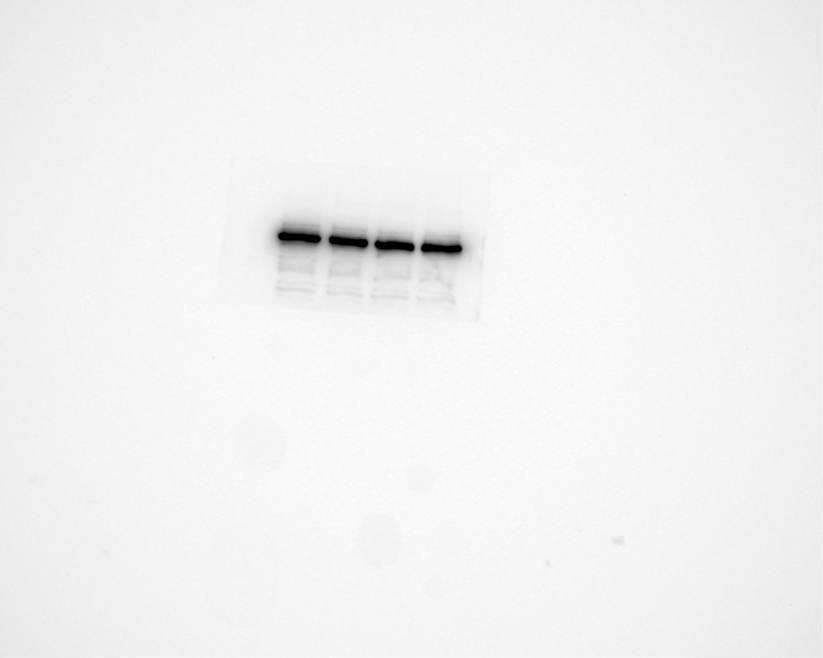


Figure 7B

NF-kB p65


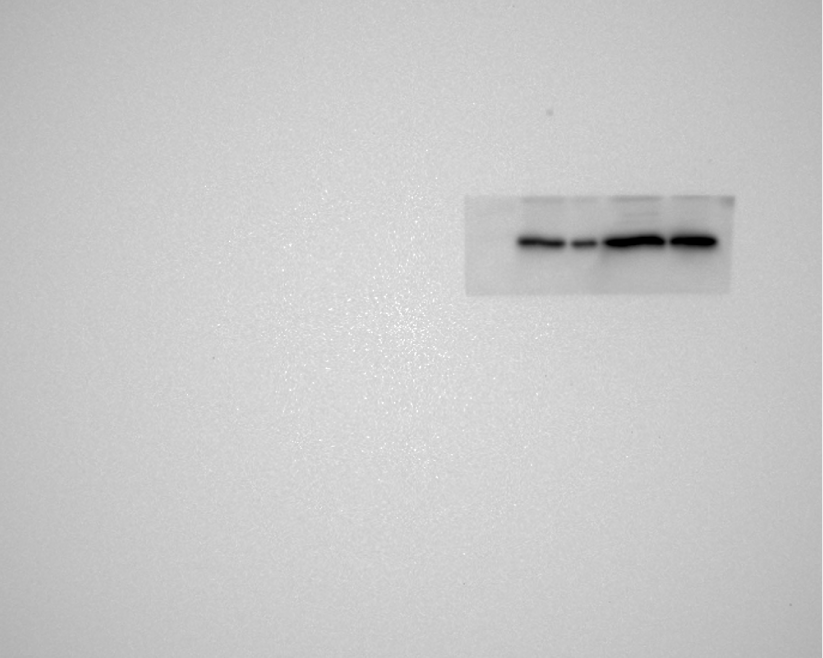


β-actin


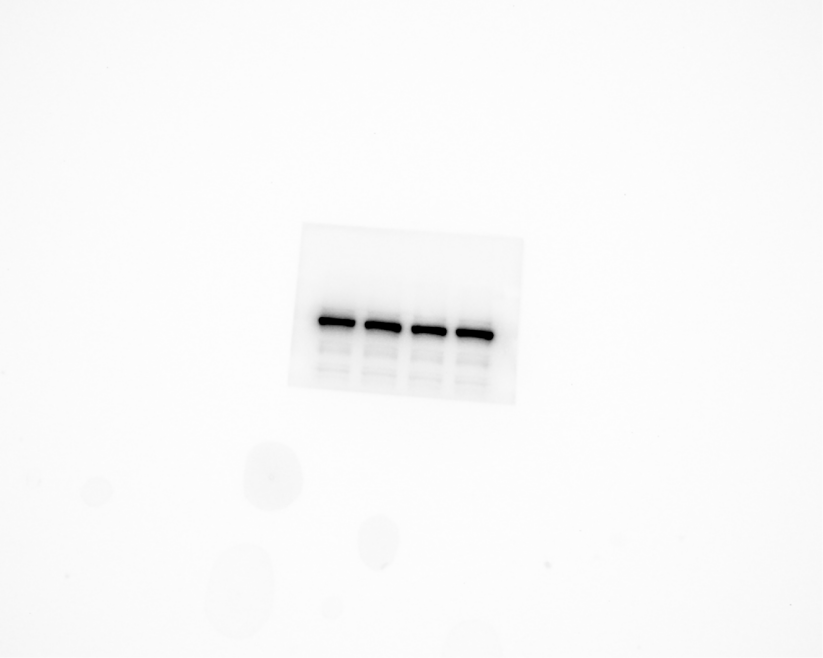


NF-kB p65


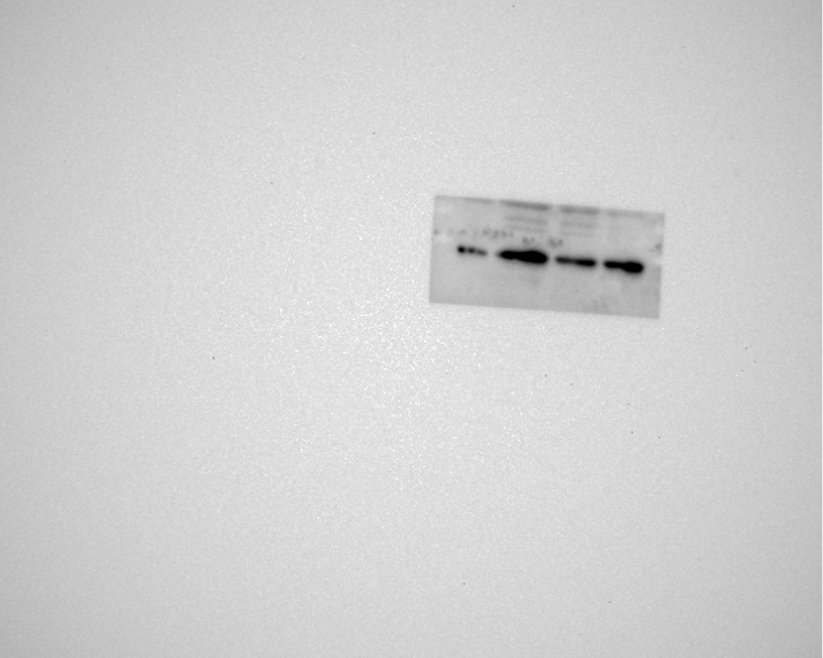


Lamin B1


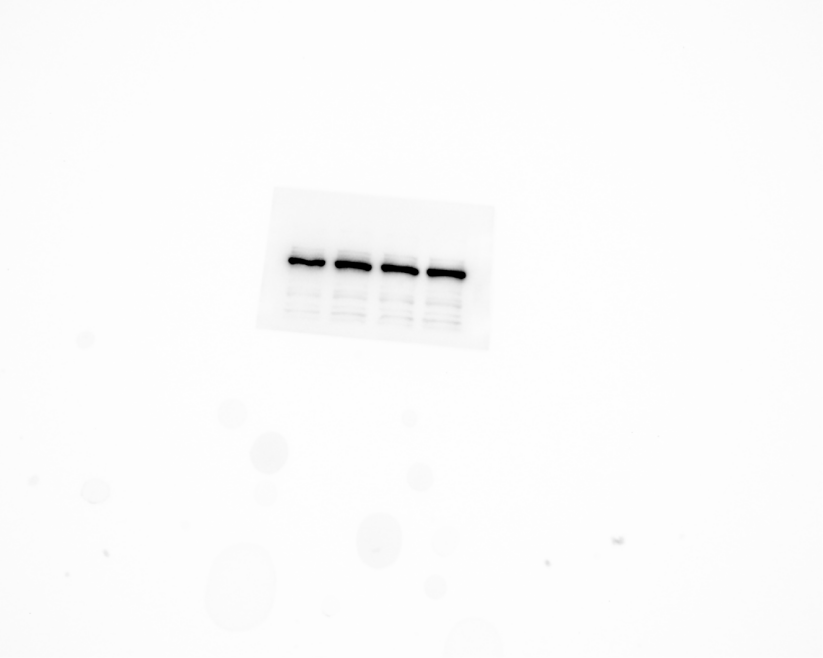


Figure 9A

p-NF-kB p65


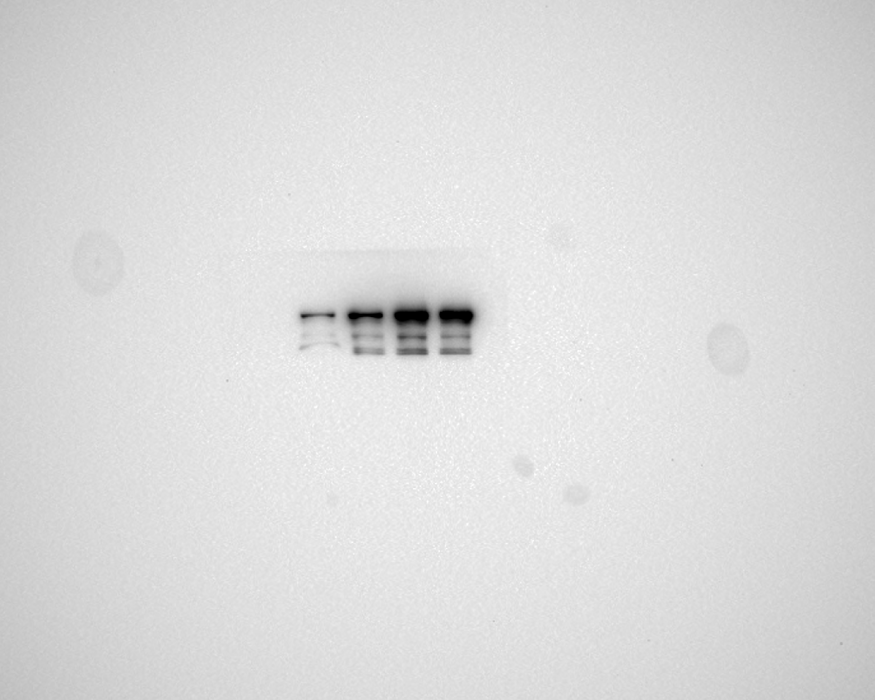


NF-kB p65


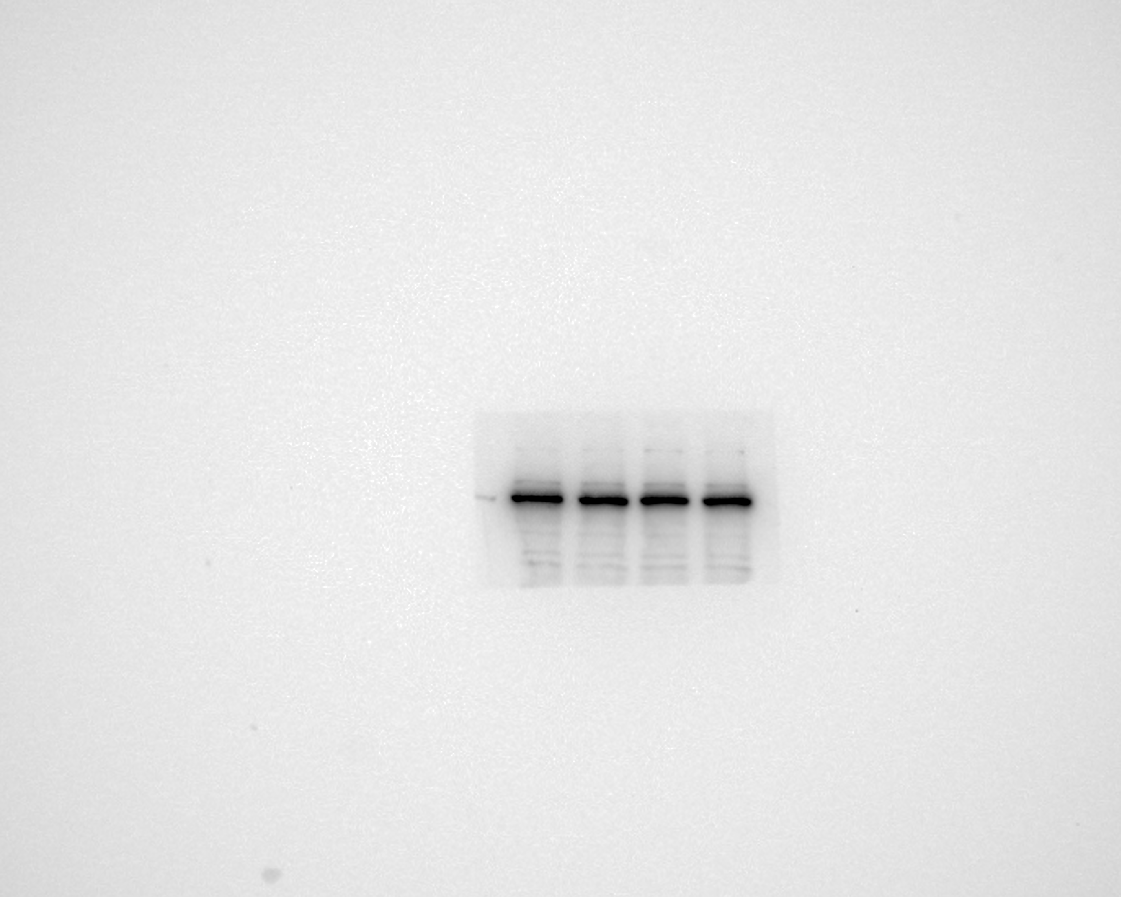


p-IκBα


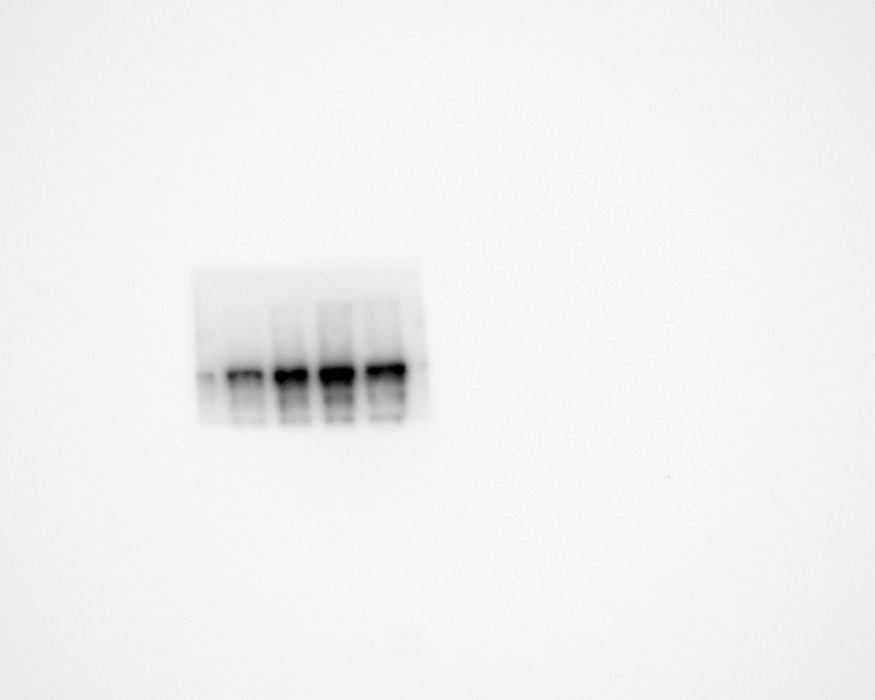


IκBα


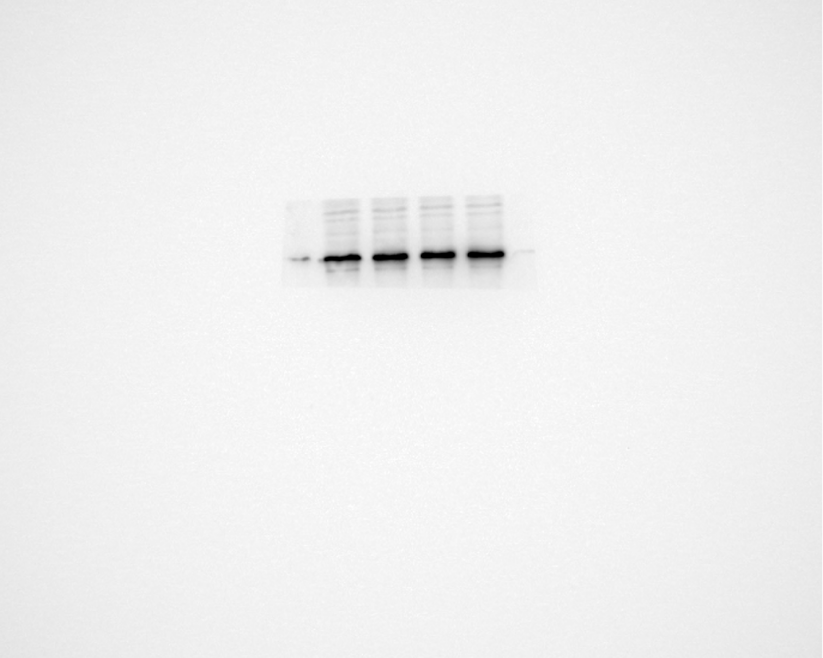
、

β-actin


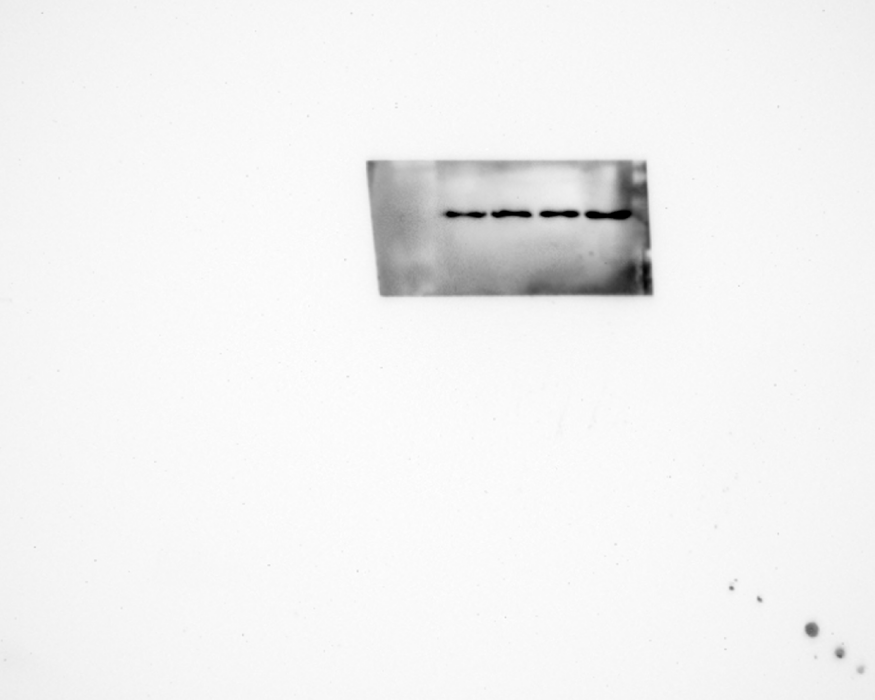

Supplement: Supplementary file 1 — Supplementary Material 1: Supplementary Figure 1. Merging and de-batching of three PD-related GEO datasets A. Density distribution comparison before (up) and after (down) batch removal. B. UMAP distribution comparison before (up) and after (down) batch removal. C. Comparison of data distribution before (up) and after (down) batch effect removal. Supplementary Figure 2. Identification and enrichment analysis of downstream targets of CD. A. The Venn diagram of CD targets in TargetNet, SymMap, SwissTargetPrediction, HERB 2.0 and ITCM databases. B. Cytoscope 3.9.1 software was used to construct the network diagram of CD and the targets. The green rhombus nodes represent CD, and the purple oval nodes represent the targets. C. The bar chart shows the top 10 results of the three items of biological process, cellular component and molecular function in the GO enrichment analysis. D. The bubble chart shows the top 20 items of the KEGG enrichment analysis results. Supplementary Table 1. The functional enrichment analysis results of the 31 genes in the intersection of DEGs in PD and IRGs. Supplementary Table 2. Targets of RELA predicted TRRUST, hTFtarget, ENCODE, JASPAR and MotifMap databases. Supplementary Table 3. The functional enrichment analysis results of RELA targets. Supplementary Table 4. The ingredients of ITCM and HERB 2.0 databases. Supplementary Table 5. Prediction of drug-likeness and ADMET properties of the potential drugs. Supplementary Table 6. The targets of Cardamonin in TargetNet, SymMap, SwissTargetPrediction, HERB 2.0 and ITCM databases. Supplementary Table 7. The functional enrichment analysis results of 104 targets of CD. [file 41065_2026_660_MOESM1_ESM.zip › WB_raw image file.docx]
